# Applicability of open science practices to completed research projects from different disciplines and research paradigms

**DOI:** 10.12688/f1000research.111383.2

**Published:** 2022-07-20

**Authors:** Jürgen Schneider

**Affiliations:** 1 University of Tübingen, Tübingen, Germany

**Keywords:** Open Science, Open Access, Open Data, Open Education, Open Evaluation, Open Methods, Open Participation, Open Policies, Open Software, Open Tools

## Abstract

The purpose of this data collection was to uncover the extent to which communities have emerged that cultivate a shared understanding of open science. In a cross-sectional survey, we assessed the applicability of 13 open science practices over different disciplines and research paradigms. Focusing on completed research projects, participants were able to draw informed evaluations concerning the applicability of open science practices. The total sample is N=295 researchers, with approximately equal numbers from six broad disciplines (between 42 and 52 participants per discipline). The survey included an attention check.

## Introduction

“Open science” is not a new term in the repertoire of academic disciplines, it has developed over the last several decades. However, what researchers understand by open science and which open science practices they refer to in particular varies greatly between research projects. In some research projects, open science practices are closely related with aspects of the research paradigm’s philosophy of science (e.g., preregistration in critical rationalism), in other research projects, they are at odds (e.g., replicability in constructivism). Accordingly, the implementation of open science practices naturally varies in degree of implementation and stage of development. The purpose of this data collection was to reveal the extent to which subcommunities have emerged that share a common profile of implementing open science practices and how they are related to research paradigms and disciplines.

## Materials and methods

### Data collection protocol

The study was conducted as a cross-sectional survey using the survey tool *formr* ( Arslan, Walther & Tata, 2020). In order to achieve a high ecological validity, the survey referred to the applicability of open science practices in concrete research projects instead of time periods (e.g., referring to the past year). We focused on evaluating *completed* research projects as participants were able to draw on their experiences from all phases of the project, which allowed them to make informed assessments of the factors that influenced research project decisions. Participants were recruited via the online access panel provider prolific.co. We used prolific’s built in filter to target researchers (“Industry Role = Researcher”). In the study description for prolific, we merely indicated that the study addressed practices in research projects; the focus on open science practices was avoided to reduce selection bias in the sample:

“In this study, you will indicate whether 13 practices are *potentially applicable* to a research project you were conducting. The survey contains *only 16 items* in total.

We are looking for participants who have conducted a *research project* associated with *one* of the *disciplines*

• Natural sciences
• Engineering and technology
• Medical and health sciences
• Agricultural and veterinary sciences
• Social sciences
• Humanities and the arts”

Participants took on average 3.98 minutes (median 3.43) to answer the survey and received USD 0.85 as compensation (approx. USD 12.81 per hour on average). The first participant started on August 20 2021, the last session on March 16 2022.

We aimed for a sample distributed across all research disciplines. For this reason, we drew on the classification of research fields from the OECD Frascati Manual (OECD, 2015). The Frascati Manual is an internationally acknowledged standard on the methodology of collecting and using research and development statistics, developed by the OECD. As a standard, it is the first choice for the definition and taxonomy of research disciplines. For each “broad classification” from the manual (natural sciences, engineering and technology, medical and health sciences, agricultural and veterinary sciences, social sciences, humanities and the arts) we aimed for n=50 participants, which would have led to a total sample of N=300 (limit of allocated financial resources). As soon as 50 participants from a discipline (broad classification) finished the survey, access to the survey closed for participants from that discipline (“cell closed”). For two broad classifications we exceeded the stopping rule (Natural Sciences, Social Sciences), as cells only closed after the last participant from that cell *finished* the survey, while further participants from that cell were still able to begin the survey (see Table 1). In addition, we were only able to recruit 42 participants for the agricultural and veterinary sciences despite several postings on prolific. This may not be surprising, since agricultural and veterinary sciences is a narrower field compared to the other broad classifications. This is also the reason why the data collection is spread over a longer period of time. After the start of data collection, participants of the other broad classifications could be collected within a few weeks. Due to the repeated invitation of researchers from the agricultural and veterinary sciences, the period of data collection stretched out, unfortunately only a few additional participants could be recruited (see codebook).

**Table 1.** Count of participants from each discipline.

Discipline (broad classification)	Count of participants
Natural sciences	52
Engineering and technology	50
Medical and health sciences	50
Agricultural and veterinary sciences	42
Social sciences	51
Humanities and the arts	50
**∑**	**295**

### Procedure and measures

On the first page, participants agreed to the declaration of consent.

On the second page they indicated the *discipline* in which the research project was based regarding which they would like to answer the following questions: “Discipline. On the next page you will answer questions regarding a previous research project. To which discipline is this research project most closely related?” In a dropdown menu, participants were able to choose from all 42 second-level classifications from the OECD Frascati Manual (OECD, 2015). In using the second-level classifications, we tried to avoid inconsistent assignments to the broad classifications by the participants. After that an attention check item was displayed (see below).

The third page gave a quick instruction on how to answer the items following on the next page: “When answering the items on the next page, please think of a research project of yours that you have already completed. Regardless of whether you actually applied the practices in this research project: Which of the practices would have been *potentially applicable*, given all the characteristics and circumstances of the project? This includes both scientific, and practical considerations in conducting the study.”

On top of the fourth page the following question was displayed: “To what extent are the following behaviors applicable in your research project?” Which was then followed up by 13 items on open science practices (item labels see Table 2). The practices were derived and synthesized using a top-down and bottom-up approach from the FOSTER Taxonomy of Open Science (top-down) and nine additional expert interviews from different disciplines (bottom-up). Through the top-down and bottom-up approach, blind spots were mutually exposed to ensure that the broadness of open science practices are reflected in the survey. The FOSTER taxonomy is the only taxonomy on open science that we know of. It was created as part of the FOSTER Plus project, an EU-funded project on Open Science. The goals in the project explicitly covered the generation of high quality training resources, which includes the taxonomy we use. For the bottom-up approach, we interviewed nine experts in open science. We recruited the experts from an open science fellows program in which they served as mentors. Following the theoretical sampling approach, we recruited mentors who came from a variety of disciplines (e.g., sociology, computer science, sinology) and applied different research paradigms (qualitative, quantitative, mixed methods, theoretical). In a focused interview, interviewees were given a narrative prompt to retrospectively consider open science practices in their field: “Please recall one of your most recently completed research projects. Thinking about the entire span of the project, from the initial idea to the completion of the project, what aspects of open science do you consider significant and how can they be exemplified in research projects?” The interviewer then asked follow-up questions about other practices: “Are there other aspects of Open Science that you consider significant in your research projects (i.e., potentially others as well)? If so, how could these be implemented?”. The interviewer also asked follow-up questions to clarify individual practices mentioned. Two trained coders transcribed and segmented the interview material around each open science practices mentioned. Disagreements in the coding process were resolved through discussion throughout the coding process. With the segmented material, the coders conducted a qualitative content analysis. In two stages they abstracted the practices named by the interviewees to an equivalent level of abstraction. These practices thus obtained were finally compared and synthesized with the FOSTER taxonomy resulting in 13 items on open science practices.

On the bottom of the page we assessed the research paradigm the project was situated in: “Research paradigm. What was the project’s primary research interest and design?” with the single choice answer categories “mainly qualitative empirical”, “mainly quantitative empirical”, “explicitly mixed-methodological (equally qualitative and quantitative empirical)” and “nonempirical”.

**Table 2.** The items of the 13 open science practices addressed in the survey.

**Involving the non-academic public in the research process** The non-academic public is involved in the process of scientific research – whether in community-driven research or global investigations. Citizens do scientific work—often working together with experts or scientific institutions. They support the generation of relevant research questions, the collection, analysis or description of research data and make a valuable contribution to science. *“Citizen Science”*
**Publicly sharing project plans to encourage feedback and collaboration** Researchers make their project plans publicly available at an early stage (e.g., on social media, websites) to optimize the study design through feedback and to encourage collaboration. *“Open Collaboration”*
**Preregistering study plans** Researchers submit important information about their study (for example: research rationale, hypotheses, design and analytic strategy) to a public registry before beginning the study. *“Preregistration”*
**Publicly sharing the methodology of the research process** Researchers describe methods, procedures and instruments that are used in the process of knowledge generation and make them publicly available. *“Open Methodology”*
**Using Open file formats and research software** Researchers use software (for analysis, simulation, visualization, etc.) as well as file formats that grant permission to access, re-use, and redistribute material with few or no restrictions. *“Open File Formats and Research Software”*
**Publicly sharing research materials** Researchers share research materials, for example, biological and geological samples, instruments for measurement or stimuli used in the study. *“Open Materials”*
**Publicly sharing data analyses** Researchers make the procedure of the data analyses and their scripts (“code”) publicly available so that others are able to reach the same results as are claimed in scientific outputs. *“Open Code/Open Script”*
**Publicly sharing research data** Researchers publicly provide the data generated in the research process free of cost and accessible so that if can be used, reused and distributed provided that the data source is attributed. *“Open Data”*
**Generating open educational resources** Researchers produce and release teaching, learning and research materials in any medium that reside in the public domain or have been released under an open license that permits no-cost access, use, adaptation and redistribution by others with no or limited restrictions. Open educational resources include full courses, course materials, modules, textbooks, streaming videos, tests, images, software, and any other tools, materials, or techniques used to support access to knowledge. *“Open Educational Resources”*
**Deciding for openness in the peer review process** Researchers opt for some kind of openness in the peer review process, including making reviewer or author identities open, publishing review reports or enabling a broader community to participate in the process. *“Open Peer Review”*
**Publishing open access** Researchers publish their research paper online, free of cost with free reusability regarding copyright restrictions. This involves any form of open access (preprints, gold and hybrid open access, etc.). *“Open Access”*
**Providing open source code of software** Researchers make source code for a piece of software that was developed in the research process publicly available, along with an open source license permitting reuse, adaptation, and further distribution. *“Open Source”*
**Communicating research results to nonacademics** Researchers use appropriate skills, media, activities, and dialogue to produce one or more of the following personal responses to science: Awareness, Enjoyment, Interest, Opinions, Understanding. Science communication may involve science practitioners, mediators, and other members of the general public, either peer-to-peer or between groups. *“Science Communication”*

Note: The item format was a 4-point Likert scale with the answer format from “not applicable at all” to “highly applicable” on the question “To what extent are the following behaviors applicable in your research project?”.

For details on items and item statistics, see the codebook (created with the R package *codebook*; Arslan, 2019) in the *Extended data* ( Schneider, 2022).

### Data validation

Participants had to pass an attention check at the beginning of the survey in order to be able to complete the other questions. The attention check looked as follows:

“ *Please read the following scenario briefly and answer a question about it:*

A famine has broken out in your village. You and some others have been chosen to leave the village and search for food. It begins to rain heavily and soon there will be flooding. Participants in studies like this are sometimes not very attentive. We have included this question here to check if you have actually read the scenario. If you read this, leave the following question unanswered just click next.

*According to the scenario, would it be appropriate to take the raft and leave the others behind?*”

Followed by a seven-point Likert scale with the ankers “absolutely no” and “absolutely yes”. The attention check was considered “passed” if *nothing* was marked on the seven-point Likert scale (i.e. an NA value on this item). Overall, 20 participants eligible for participation failed the attention check and were thus excluded. These participants are not included in the data set that is available for download ( Schneider, 2022). They jumped to the end of the survey after failing the attention check and therefore did not complete the 13 items on the open science practices.

As a limitation regarding data validation, it should be noted that we did not target a representative sample of researchers across disciplines. For the data set, it was important that we had variance in the backgrounds of the researchers. Any analyses comparing disciplines should therefore be interpreted with caution.

### Ethical approval and consent to participate

The present data collection received approval from the ethics committee of the Faculty of Economics and Social Sciences at the University of Tübingen (no approval number). Participants agreed to the consent details printed below before beginning the survey.

### Future analyses

In the future, the data will be analyzed to answer the questions whether there are different communities in the application of open science practices and to what extent the open science practice profiles of these communities are similar or different to each other. Are there open science practices that all communities share? Are there practices for which there are particularly strong differences between communities? In addition, the role of research disciplines and research paradigms will be explored.

## Data availability

### Underlying data

Zenodo: Applicability of open science practices to completed research projects from different disciplines and research paradigms. https://doi.org/10.5281/zenodo.6834569 ( Schneider, 2022).

This project contains the following underlying data:

• osc_data.RData (data set as RData-file)
• osc_data.csv (data set as CSV-file)

### Extended data

Zenodo: Applicability of open science practices to completed research projects from different disciplines and research paradigms. https://doi.org/10.5281/zenodo.6834569 ( Schneider, 2022).

This project contains the following extended data:

• codebook.html (codebook report of survey and its items)
• STROBE-checklist-v4-cross-sectional.pdf (STROBE Statement: Checklist of items that should be included in reports of cross-sectional studies)
• Consent Statement.pdf (Consent Statement: Details of the Consent Statement the participants agreed to)

Data are available under the terms of the Creative Commons Attribution 4.0 International license (CC-BY 4.0).
