# Supplementary material for: Applicability of open science practices to completed research projects from different disciplines and research paradigms
Source: F1000Res. 2022 Jul 20;11:408. Originally published 2022 Apr 11. [Version 2] doi: 10.12688/f1000research.111383.2 (PMC9391739; doi:10.12688/f1000research.111383.2)
Supplement: Peer review history

# Peer review: Reviewer response for version 2

**DOI:** 10.5256/f1000research.136169.r145712

**Published:** 2022-09-26

**Authors:** Henrik Bellhäuser

**Affiliations:** 1 Department of Psychology, Mainz University, Mainz, Germany

In this data note, the author gives access to and describes a data set collected from N=295 researchers from various fields that assessed whether a number of open science practices were applicable in their discipline.

 I value the approach to make the data accessible and overall I think the author did a very good job in documenting all details relevant for this data collection. The data is both interesting and easily reusable based on this data note.

 I have only few minor comments as I believe the previous reviewers and the author already solved the major issues:

 - At first, I thought the declaration of consent would not be included. Later, I found it on Zenodo—you might want to mention on p.4 where the reader can find it.

 - If possible, you might add the link to Zenodo already in the abstract.

 - The codebook is great—makes me want to start form as well. If it would be possible to adjust the y-axes of all variables so that the size of the bars are comparable, it would be even better in my opinion.

Are sufficient details of methods and materials provided to allow replication by others?

Yes

Is the rationale for creating the dataset(s) clearly described?

Yes

Are the datasets clearly presented in a useable and accessible format?

Yes

Are the protocols appropriate and is the work technically sound?

Yes

Reviewer Expertise:

educational psychology

I confirm that I have read this submission and believe that I have an appropriate level of expertise to confirm that it is of an acceptable scientific standard.

# Peer review: Reviewer response for version 2

**DOI:** 10.5256/f1000research.136169.r146059

**Published:** 2022-08-19

**Authors:** Ke Gong, Chuang Liu, Xiangtong Jia

**Affiliations:** 1 China Academy of Science, Beijing, China; 2 College of Life Sciences/Center for Genomics and Bio-computing, North China University of Science and Technology, Tangshan, China; 3 Nankai University, Tianjin, China

The research involves nearly all aspects of open science practices (including six broad disciplinary), the span of disciplines is large, the content is complex and the data is valuable. The work had been approved by an ethics committee and the data had been validated properly.

 However, the paper does not mention similar research but no comparable analysis. 

 The total number of the validated participants is 295, and 42-52 for each discipline. It is suggested to increase the number of participants and consider to have different number of participants for different disciplines according to the proportion of research projects of related discipline, so that it may be more reasonable.

 The practice of open science involves complex socio-technical elements, including scientific communities, general public and companies and so on, participants with different backgrounds may have different opinions, this might be further considered.

 I agree very much for the setting of attention check, to ensure the reliability of the survey and to avoid the random answers of the respondents. The questionnaire form could be concise and friendly, can even add some bogus items options.

 Furthermore, I wonder if the author has considered privacy issues during the survey, how have these issues been treated? The survey asked participant to make a judgement between 1-4, why not chosen between 1-10?

Are sufficient details of methods and materials provided to allow replication by others?

Yes

Is the rationale for creating the dataset(s) clearly described?

Yes

Are the datasets clearly presented in a useable and accessible format?

Partly

Are the protocols appropriate and is the work technically sound?

Yes

Reviewer Expertise:

Electronic engineering and telecommuication, engineering education, scientific policies

We confirm that we have read this submission and believe that we have an appropriate level of expertise to confirm that it is of an acceptable scientific standard.

# Peer review: Reviewer response for version 2

**DOI:** 10.5256/f1000research.136169.r144742

**Published:** 2022-08-01

**Authors:** Suzanne Dumouchel

**Affiliations:** 1 TGIR Huma-Num, CNRS, Paris, France

I have read the answers to my review and seen the additions and it's fine for me. I approve the publication.

Are sufficient details of methods and materials provided to allow replication by others?

Partly

Is the rationale for creating the dataset(s) clearly described?

Yes

Are the datasets clearly presented in a useable and accessible format?

Yes

Are the protocols appropriate and is the work technically sound?

Yes

Reviewer Expertise:

NA

I confirm that I have read this submission and believe that I have an appropriate level of expertise to confirm that it is of an acceptable scientific standard.

# Peer review: Reviewer response for version 1

**DOI:** 10.5256/f1000research.123102.r137032

**Published:** 2022-05-27

**Authors:** Lauren Cadwallader

**Affiliations:** 1 Public Library of Science, San Francisco, CA, USA

This research aims to gain insights into open science practices in six broad disciplinary areas by asking researchers about the potential to practice open science behaviours in relation to a past project. Thirteen different open science behaviours are included, which give a comprehensive view of open science rather than just publishing related activities.

 The rationale for the study is good and asking researchers about past projects, rather than asking them to predict future work, is an interesting and valuable approach to assessing open science practices. The range of open science practices included in the survey is broad, which should give a good insight into all activities that contribute to the aims of being open. However, one aspect I think is lacking is the extent to which researchers were mandated by their funder or institution to carry out any of these activities, which would add context to the extent to which they view certain practices as applicable.

 In terms of the demographics of the survey sample, it is good to see balanced sample sizes in the disciplines and the inclusion of whether the researcher's work is quantitative, qualitative or mixed methods. The methodology should include more information on which geographical areas were targeted for respondents and information on career stages of the respondents if possible, as these are both factors in adoption of open science practices.

 I have concerns that the wording used in the Likert scale may have been confusing for respondents. The aim was to get researchers to report on their past research but the question about open science practices was in the present tense ("To what extent ***are*** the following behaviours applicable..." [emphasis added]). I'm also unsure of how to interpret the "not at all applicable" response - the codebook seems to suggest a "N/A" option was also offered but this doesn't seem to have been used by anyone. For example, were all respondents creating software but not all thought that sharing software openly applied to their project? Or were the respondents who chose "not at all applicable" really saying they didn't create software? Including the survey instrument (i.e. a pdf copy of the survey questions as presented to the respondents) might help with understanding this better.

 A couple of other specific points:

 - There is no mention of data handling with respect to GDPR rules.

 - The codebook gives an identical explanation for two variables - osp_sco and osp_cit. Presumable the latter is Citizen Science and not Science Communication.

 The dataset in Zenodo is well presented and easy to use (aside from the labelling mistake mentioned above). As mentioned earlier, I would recommend adding the survey instrument to the dataset to aid understanding and also enable others to repeat this approach.

Are sufficient details of methods and materials provided to allow replication by others?

Partly

Is the rationale for creating the dataset(s) clearly described?

Yes

Are the datasets clearly presented in a useable and accessible format?

Partly

Are the protocols appropriate and is the work technically sound?

Yes

Reviewer Expertise:

Scholarly communications, publishing, open science.

I confirm that I have read this submission and believe that I have an appropriate level of expertise to confirm that it is of an acceptable scientific standard, however I have significant reservations, as outlined above.

# Response

**Published:** 2022-07-14

**Authors:** Jürgen Schneider

**Affiliations:** University of Tübingen, Germany

Thank you as well for the constructive comments. I will respond to your comments chronologically:

1. We agree that it would have been interesting to have data on the mandates of funders or institutions. Unfortunately, we can't collect this data a posteriori. However, there is first evidence that mandates at the level of the research institution do not necessarily have the greatest impact on researchers' open science practices. For example, the existence of a research data policy at a research institution is not related to higher data sharing: https://doi.org/10.31234/osf.io/9yhcz
2. We agree that information on the geographical areas and career stage of participants would complement the data set. Unfortunately, we had to keep the survey short due to financial resources. Therefore, we don’t have these data.
3. I agree that the wording of the items in the present tense is unfortunate. Participants were encouraged to ask us questions via the survey-internal direct messaging system if any questions arose during the course of the questionnaire. The participants did indeed use this opportunity, and we received 27 direct messages. However, these inquiries were all aimed at the attention check that was placed at the beginning of the questionnaire, no questions arose about the subsequent items.
4. We have been careful not to collect personalized data in the survey for which the GDPR would be relevant.
5. Thank you for the pointing out the mistake in the codebook. We corrected this and uploaded a new version of the codebook.

# Peer review: Reviewer response for version 1

**DOI:** 10.5256/f1000research.123102.r137033

**Published:** 2022-05-24

**Authors:** Suzanne Dumouchel

**Affiliations:** 1 TGIR Huma-Num, CNRS, Paris, France

The process of the survey is clearly described such as the tools that have been used and the reasoning behind them. The selection of the researchers is also well explained and the average per discipline is relevant for this kind of study. However, it could be interesting to be precise with any information regarding the geographic coverage of the researchers and their career stage. It can contribute to better understand of the OS practices. This might have been taken into account however it is not mentioned in the paper.

 It seems particularly relevant to offer the possibility to specify the discipline and not to stay at the level of the broad classification. On this topic, further explanation of the reasoning behind the broad classification could be useful, especially to understand why "agricultural and veterinary sciences" are not part of natural sciences. What is the reasoning behind this?

 The selection of the Frascati Manual seems to be relevant but more details can be provided to mention maybe other options and to explain why this one has been selected and upon which criteria?

 This precision can be expected as well for the FOSTER Taxonomy of Open Science.

 The role of the nine additional expert interviews is not clear enough. It is difficult for the reader to understand how practices have been synthesised from both the FOSTER Taxonomy and the expert interviews. The paper could be stronger by explaining 1) the goal and process of the interviews; 2) the selection of these experts (for instance regarding the disciplines as it is the main focus in the paper); 3) how the practices have been built from these interviews and the taxonomy. For instance, it can be interesting to highlight the potential bias or reasoning to synthesise in one way or another.

 It is interesting to see that the survey has run between August 2020 and March 2022. Regarding the increase of knowledge and practices related to Open Science, the answers might have evolved between both. Even if it can be quite tricky to determine this aspect, it can be relevant to mention or highlight any difference if some have been noticed. 

 Lastly the reader could expect a short conclusion about the main outputs of the survey. And if the goal is to provide it in a dedicated paper, some highlights can be useful or at least it can be announced at the end of the paper. 

 In any case, this kind of survey is particularly relevant and should contribute to implement the OS practices in the different disciplines as well as to better identify the needs for the researchers. With these small precisions, we believe the paper (and the data collected) contributes to the OS reflexion.

Are sufficient details of methods and materials provided to allow replication by others?

Partly

Is the rationale for creating the dataset(s) clearly described?

Yes

Are the datasets clearly presented in a useable and accessible format?

Yes

Are the protocols appropriate and is the work technically sound?

Yes

Reviewer Expertise:

research infrastructures, social sciences and humanities, FAIR data, EOSC, Open Science

I confirm that I have read this submission and believe that I have an appropriate level of expertise to confirm that it is of an acceptable scientific standard, however I have significant reservations, as outlined above.

# Response

**Published:** 2022-07-14

**Authors:** Jürgen Schneider

**Affiliations:** University of Tübingen, Germany

Thank you very much for your constructive feedback. I will respond to your comments chronologically:

1. We agree that information regarding the geographic coverage and their career stage would complement the data set. Unfortunately, we had to keep the survey short due to financial resources. Therefore, we don’t have these data.
2. We agree that specifying the disciplines might be a valuable information for data users. Therefore, we have now included the disciplines in the data set as well as the Codebook.
3. Concerning the Frascati manual, we added the following paragraph to the methods section: “The Frascati Manual is an internationally acknowledged standard on the methodology of collecting and using research and development statistics, developed by the OECD. As a standard, it is the first choice for the definition and taxonomy of research disciplines.”
4. Concerning the FOSTER taxonomy, we added the following paragraph to the methods section: “The FOSTER taxonomy is the only taxonomy on open science that we know of. It was created as part of the FOSTER Plus project, an EU-funded project on Open Science. The goals in the project explicitly covered the generation of high quality training resources, which includes the taxonomy we use.”
5. We agree that more information on the synthesis of the FOSTER taxonomy and the interviews would help readers understand the construction of the survey. We therefore added the following paragraph to the methods section: “Through the top-down and bottom-up approach, blind spots were mutually exposed to ensure that the broadness of open science practices are reflected in the survey. […] For the bottom-up approach, we interviewed nine experts in open science. We recruited the experts from an open science fellows program in which they served as mentors. Following the theoretical sampling approach, we recruited mentors who came from a variety of disciplines (e.g., sociology, computer science, sinology) and applied different research paradigms (qualitative, quantitative, mixed methods, theoretical). In a focused interview, interviewees were given a narrative prompt to retrospectively consider open science practices in their field: "Please recall one of your most recently completed research projects. Thinking about the entire span of the project, from the initial idea to the completion of the project, what aspects of open science do you consider significant and how can they be exemplified in research projects?" The interviewer then asked exmanent follow-up questions about other practices: "Are there other aspects of Open Science that you consider significant in your research projects (i.e., potentially others as well)? If so, how could these be implemented?". The interviewer also asked intrinsic follow-up questions to clarify individual practices mentioned. Two trained coders transcribed and segmented the interview material around each open science practices mentioned. Disagreements in the coding process were resolved through discussion throughout the coding process. With the segmented material, the coders conducted a qualitative content analysis. They abstracted the practices named by the interviewees to an equivalent level of abstraction in two stages. These practices thus obtained were finally compared and synthesized with the FOSTER taxonomy resulting in 13 items on open science practices."
6. We agree that the choice of the timeframe may raise some questions. The reason for the long time frame was our desire to recruit more researchers from the broad classification of "agricultural and veterinary sciences". Accordingly, we have added a paragraph: “This is also the reason why the data collection is spread over a longer period of time. After the start of data collection, participants of the other broad classifications could be collected within a few weeks. Due to the repeated invitation of researchers from the agricultural and veterinary sciences, the period of data collection stretched out, unfortunately, only a few additional participants could be recruited (see codebook).”
7. We agree that information on the output of the survey would be interesting information for the readers. We included the following paragraph in a newly created “Future analyses” section: “In the future, the data will be analyzed to answer the questions whether there are different communities in the application of open science practices and to what extent the open science practice profiles of these communities are similar or different to each other. Are there open science practices that all communities share? Are there practices for which there are particularly strong differences between communities? In addition, the role of research disciplines and research paradigms will be explored.”
